# Blood purification for sepsis: an overview

**DOI:** 10.1093/pcmedi/pbab005

**Published:** 2021-02-25

**Authors:** Ling Zhang, Yuying Feng, Ping Fu

**Affiliations:** Division of Nephrology, Kidney Research Institute, West China Hospital of Sichuan University, Chengdu 610041, China; Division of Nephrology, Kidney Research Institute, West China Hospital of Sichuan University, Chengdu 610041, China; Division of Nephrology, Kidney Research Institute, West China Hospital of Sichuan University, Chengdu 610041, China

**Keywords:** sepsis, acute kidney injury, blood purification

## Abstract

Sepsis is a life-threatening organ failure exacerbated by a maladaptive infection response from the host, and is one of the major causes of mortality in the intensive care unit. In recent decades, several extracorporeal blood purification techniques have been developed to manage sepsis by acting on both the infectious agents themselves and the host immune response. This research aims to summarize recent progress on extracorporeal blood purification technologies applied for sepsis, discuss unanswered questions on renal replacement therapy for septic patients, and present a decision-making strategy for practitioners.

## Introduction

In intensive care units (ICU), septic disease is the most common causes of death. There are approximately 19.4 million cases worldwide, with potentially 5.3 million deaths annually.^1^ Taking into account the third international consensus for sepsis and septic shock (Sepsis-3), the definition of sepsis was revised in 2016 as “organ dysfunction, which is life-threatening, caused by an infected host”.^2,3^ Identification of organ dysfunction in infected patients may be assisted by use of the rapid sequential organ failure assessment (SOFA) score, in which a score of ≥2 points suggests sepsis^2,3^ and is linked with in-hospital mortality of 10%.^4,5^

Septic shock is now defined as “sepsis with vasoactive therapy requirement that medium arterial pressure be maintained as much as 65 mm Hg and lactate height as > 2 mmol/L despite sufficient volume reactivation”.^3^ This new definition arose from expanded understanding of sepsis pathophysiology, management, and epidemiology since the previous revision in 2001,^3,6^ and highlights the significant role of adaptive and protective homeostatic/allostatic response during sepsis.^7^

Conventional septic shock management includes antibiotics, symptomatic support for organ dysfunction, and surgery to contain the infection source if required. Despite recent advances in intensive care, mortality can reach 40% at day 28 in cases of septic shock.^8^ Thanks to technological advances in extracorporeal circuits and membranes, we have developed more options regarding adjuvant therapy for septic shock. Various methods of blood purification have been used and researched in recent decades by modulating sepsis-inducing immune reactions. However, these technologies remain a point of discussion until their clinical effectiveness can be verified by further positive multicenter randomized controlled trials (RCTs).^9^

This review will summarize current literature on available extracorporeal blood purification techniques for sepsis, discuss unanswered questions on RRT for septic patients, and present a decision-making strategy for medical practitioners.

## Septic immune response pathophysiology and blood purification Rational

Immune system identification of a pathogen is considered the primary immune reaction for sepsis. Molecular patterns (PAMPs), including lipopolysaccharides (LPS), lipoteichoic acid, DNA or RNA fragments, flagellin and mannan, as part of the infection, are detected by pattern recognition receptors (PRRs) displayed on the membranes of the immune cells.^10^ This signal activates leukocyte activation and the development of both proinflammatory and anti-inflammatory cytokines, such as interleukin-1, IL-6, and tumor necrosis factor-alpha (TNF-a). The massive systemically dysregulated cytokine response, referred to as a “cytokine storm”, is usually considered to be the key pathophysiological response that leads to organ dysfunctions.^11–13^ Damage associated molecular patterns (DAMP), including high-mobility box 1 group (HMGB1), heat-shock proteins, and histones, are expressed on the surface of wounded host cells. DAMPs can be released and recognized via PRRs, which trigger the unregulated immunoinflammatory cycle,^14^ facilitating an immune-paralysis state, and resulting in sepsis-induced deaths.^15^

Based on the understanding of the immune response mechanism during sepsis, adjuvant treatment strategies have been developed under the concept of modulating inflammatory mediators to restore a balanced immune response. A promising approach is removal of inflammatory mediators with extracorporeal blood purification approaches.^16^ From initial modalities intervening in a single step of the whole immune process, to later invented cartridge choice targeting two or more clinical issues, significant progression has been made in this field. There have been several hypotheses developed to explain the underlying mechanism. First, Ronco *et al*. proposed the “cytokine peak hypothesis”: blood purification decreases pro-inflammatory and anti-inflammatory mediator concentrations during early sepsis, avoiding attaining a “toxic threshold”, and thus limiting the local deleterious effects of cytokines and organ dysfunctions.^11^ Later, a “threshold immunomodulation hypothesis” suggested that cytokine removal from the blood would mobilize cytokines from the tissues via concentration equalization, ameliorating their local deleterious effects.^17^ More recently, the “cytokinetic model” hypothesized that as a result of a restored concentration gradient, leukocyte chemotaxis is driven towards infected tissue with higher cytokine levels by the declined cytokine blood concentrations.^16^ Finally, certain blood purification techniques may function through immune process modulation, namely the expression of surface molecules, involved in leukocyte adhesion and migration, antigen presentation, absorption of monocytes and neutrophils, and apoptosis of leukocytes.^18–21^

## Modality

### High volume hemofiltration

To improve elimination of molecules of hydrophilic middle molecular weight, high-volume hemofiltration (HVHF) was developed with a higher ultrafiltration rate (i.e. >50 ml/kg/h) than that recommended for standard kidney support for acute kidney injury (AKI).^22^ Given the complementary diffusive component, the actual ultrafiltration rate can be higher (50–70 ml/kg/h) than prescription.^23^

In spite of encouraging results in animals models,^24,25^ human studies presented inconsistent results. After numerous small-scale studies revealed better hemodynamic parameters,^26^ respiratory improvement,^27^ or a lower than expected mortality,^28–32^ some studies suggested otherwise.^33^ In 2013, a multi-center RCT, the high volume in intensive care randomized controlled (IVOIRE) trial,^34^ compared ultrafiltration flow rates of 35 and 70 ml/kg/h during a 96-hour period in 140 patients with early septic shock with AKI and did not show any difference on mortality at days 28, 60, or 90. HVHF also failed to improve secondary outcomes (RRT-, ventilator-, and vasopressor-free days; days of hospital stay; hemodynamic and standard biologic parameters; severity score evolution). This deficiency of beneficial effects was comparable to results from two meta-analyses. The first meta-analysis did not report any 28-day survival benefit of HVHF compared with conventional continuous veno-venous hemofiltration (CVVH) in septic AKI.^35^ The Cochrane collaboration meta-analysis on this subject did not conclude any beneficial effect of HVHF during sepsis compared with the usual kidney support techniques.^36^ However, in a recent RCT enrolling 82 cases, early HVHF (65 ml/kg/h for three consecutive days after burn) was reported to be beneficial by decreasing the incidence of sepsis, septic shock, duration of vasopressor treatment, and mortality in patients with severe burns. This might be the result of early clearance of inflammatory molecules and the restored immune status of patients in the HVHF group.^37^ Overall, HVHF is feasible in centers capable of providing standard continuous renal replacement therapy (CRRT); however, unwanted removal of low molecular weight molecules (especially nutrients and antibiotics) must be carefully monitored. Despite promising outcomes in earlier studies, there is no sufficient evidence to support its validity in improving primary outcomes (including patient mortality and hemodynamics).

### Cascade hemofiltration

Cascade hemofiltration was developed to avoid the significant drawbacks of HVHF mentioned above while conserving its advantages. Two hemofilters with distinct cut-off values are applied consecutively in one extracorporeal circuit: the first high cut-off hemofilter generates a first ultrafiltrate containing low and middle-weight molecules; then the second lower cut-off hemofilter clears only middle-weight molecules, while the low-weight molecules are re-injected as a predilution before the first hemofilter. In this case, cascade hemofiltration allows selective removal of middle-weight molecules.^38^

In earlier animal experiments, cascade hemofiltration decreased severity of porcine septic shock.^39^ However, in a recent study enrolling 60 patients with septic shock, no beneficial result of cascade HVHF was demonstrated compared with standard care during the first 28 days.^40^ As this was a limited data set, this offers a direction for future research.

### High cut-off membrane

The high cut-off (HCO) membrane was designed to enlarge the spectrum of middle-weight molecule removal. When applied with convective rather than diffusive modalities, the HCO membrane maximizes removal of pro-/anti-inflammatory mediators at the cost of massive albumin leakage, which could increase up to 15 g in 4 hours.^41^ Modified HCO membranes (e.g. surface or pore size homogeneity) and the choice of diffusive rather than convective modalities have been applied to achieve similar cytokine removal with acceptable albumin losses.^42,43^

The validity of HCO membranes remains controversial because early evidence comes from small RCTs and pilot studies. Research suggests that HCO membrane therapy can result in ICU mortality benefit,^44^ decreased ICU length of stay and vasopressor days,^44^ and attenuated circulating levels of inflammatory mediators (TNF-a,^45^ IL1b,^46^ IL6,^42,45^ IL8^42^ and IL10^42,45,46^) compared with standard CVVH. However, in a recent double-blind RCT enrolling 76 critically ill patients with AKI, continuous venovenous HCO failed to show any beneficial effect in reducing duration or mortality of vasopressor or albumin changes in contrast with routine treatment.^47^

### CPFA

Coupled plasma filtration and adsorption (CPFA) is a blood purification technology in which plasma is extracted from the blood by a high cut-off filter at the start of the extracorporeal circuit. The plasma is then slowly run through a sorbent cartridge where pro- and anti-inflammatory mediators and endotoxins are absorbed. The plasma filtrate is then returned to the main circuit to combine with blood, and used in standard hemofiltration. Early research into application of CPFA in sepsis suggested no benefit regarding survival or ICU length of stay but potential improvements in hemodynamics, immune function modulation, and ameliorating organ failure as opposed to HVHF.^48–54^ However, the evidence was weak as it was mainly derived from small, observational studies. A later clinical trial, COMPACT 1, incorporated filtration from plasma and adsorption.^55^ In the first 30 days of hospital mortality or clear of ICU, 192 patients were randomized to either standard care or CPFA plus standard care. COMPACT1 highlighted concerns regarding inadequate dosage, clotting risk, and cost-effective issues; however, a beneficial mortality rate was observed in a subgroup receiving the highest dose of CPFA. A subsequent trial attempted to assess the consequences of higher doses, the "combining plasma filtration and adsorption clinical trial 2" (COMPACT 2, NCT01639664). Unfortunately, because of unwanted side effects associated with the CPFA, COMPACT 2 was terminated early, and letters stating that CPFA is no longer suggested for treatment of septic shock were distributed worldwide. However, a recent retrospective study of 76 cases, indicated that CPFA safely and effectively lowered morbidity and mortality rates of patients with severe intra-abdominal infection and liver failure.^56^

### Absorptive

In recent decades, new membranes have been developed to provide kidney support together with treatment for septic shock. These membranes cope with super-high-flux membranes and present elevated absorptive capacity and enhanced clearance on middle-to-high molecular weight solutes.

#### Polymyxin B-immobilized fiber column

One of the most commonly used endotoxin removal devices is the polymyxin B-immobilized fiber column (Toraymyxin®; Toray, Tokyo, Japan). In Japan, it is commonly used for patients with serious sepsis with gram-negative bacterial infection. Recent clinical trials results remain inconclusive regarding the outcome of patient mortality using Toraymyxin®.

The validity of polymyxin B adsorption versus conventional CRRT remains inconsistent and is fiercely debated based on accumulating RCTs. Data derived from the EUPHAS trial (early application of hemoperfusion polymyxin B in abdominal septic shock)^57^ suggest a mortality benefit after baseline adjustment and a hemodynamic benefit, but no significant differences in other end points including ICU length of stay. Nevertheless, polymyxin B hemoperfusion (PMX) indicated no mortal benefits and no impact on hemodynamics and stay time in the ABDOMIX (effects of hemoperfusion with a polymyxin B peritonitis with septic shock) trial, in which PMX was assessed in 140 septic-induced peritonitis shocks.^58^ Even two retrospective studies reported by the same researcher showed conflicting results regarding 28-day mortality.^59,60^ The EUPHRATES (evaluation of the use of polymyxin B hemoperfusion in randomized controlled trials for adults treated for endoxemia and septic shock) study shared a similar result with an ABDOMIX analysis when PMX plus conventional medical treatment showed no 28-day mortality reductions in 450 eligible enrolled patients compared with conventional medical treatment alone.^61^ However, a subsequent post-hoc review on the EUPHRATES study reported that PMX had positive effects on mean arterial pressure, ventilator-free days, and mortality in subgroups of patients with septic shock and endotoxin activity (as tested in the endotoxin activity test) between 0.6 and 0.89.^62^ A recent single-center study of selective LPS adsorption using Toraymyxin® in 143 patients with sepsis after cardiac surgery showed a beneficial effect on 28-day survival.^63^ However, a recent meta-analysis^64^ (six RCTs, 857 patients) suggested no difference in mortality reduction, whereas the five previous meta-analyses^57,65–68^ demonstrated mortality benefit and supported the use of Toraymyxin® to treat patients with severe sepsis or septic shock.

To summarize, it seems that the potential beneficial effect of PMX on survival can be observed only when the control group mortality is >30%–40%.^68^ Future research should focus on patients with high expected mortality and/or EAA ≥0.6–0.89. Also, given that positive results have mainly been obtained in Japan, the genetic and enzymatic profile of patients could influence the therapy outcome.

#### CytoSorb

CytoSorb technology uses a hemoperfusion cartridge (CytoSorbents, Monmouth Junction, NJ, USA) to absorb high cytokines.^69^ In *in vivo* and *in vitro* studies, it demonstrated optimal capacity of removing broad-spectrum cytokines together with complement factors, growth factors, myoglobin, bilirubin, bile acids, PAMPs and DAMPs, with removal rates of most of the molecules >90%–95% at 120 minutes.^69–71^ However, evidence supporting its favorable outcomes on hemodynamic parameters and blood lactate levels was limited to case series.^72,73^ In an RCT comparing CytoSorb hemoperfusion with normal care (6 hours a day for 7 days), substantial elimination of cytokines during session only and no reduction in mortality or IL-6 plasma levels were observed over the course of time.^74^ As evidence of an idea, a randomized controlled experimental study of 20 patients with no need for renal replacement therapy concluded that vasopressor needs, procalcitonin (PCT), and big-endothelin-1 were reduced by more in a CytoSorb group than in a control group.^75^ CytoSorb also can be used in other conditions generating inflammation, such as severe pancreatitis or cardio-pulmonary bypass.^76,77^ The evidence supporting use of CytoSorb in septic shock remains limited. In line with preliminary clinical findings, use of CytoSorb® adsorber in real-life critically ill patients is to be documented (NCT02312024).^78^ No noteworthy declines in SOFA scores have been observed, but IL-6 levels decreased significantly after treatment.

#### HA330/380

HA330/380 (Jafron, Zhuhai City, China) are high volume resin hemoperfusion cartridges intended for patients with critical conditions incorporating a "cytokine storm". Clinical benefits of HA330 hemoperfusion reported in patients with septic shock include decreasing inflammatory mediators, mortality, and ICU length of stay, and improving hemodynamics.^79–81^ In a recent prospective observational study involving 23 patients with septic shock with AKI, the application of HA330 hemoperfusion restored CRP level and heart rate without improving prognosis.^82^

#### Oxiris

Oxiris is an AN69-based membrane designed specifically for cytokine and endotoxin adsorption alongside CRRT through surfaces treated with polyethyleneimine (PEI) and pregrafted with heparin. *In vitro* studies have found that Oxiris has a similar endotoxin adsorption to that of Toraymyxin and similar adsorption to CytoSorb for the elimination of most inflammatory mediators.^69^ Clinical study also shows a significant reduction in plasma endotoxin and inflammatory mediators after treatment with Oxiris-CRRT.^83–87^ In addition, a randomized double-blind crossover study of septic shock-related acute renal failure showed better efficacy in removal of endotoxin and inflammatory mediators by Oxiris than by normal filtering.^88^ Beneficial hemodynamic effects, mainly reflected in cardiovascular SOFA score or vasopressor dose, were also recognized universally,^89,90^ which may be associated with high effective removal of endotoxin and inflammatory mediators; however, this hypothesis remains to be confirmed. The consensus agreement from European experts regarded septic shock as the most appropriate indication for Oxiris, based on the recognition that stabilizing hemodynamic parameters is the most remarkable function of Oxiris.^91^ Additionally, significant improvement in organ function has been shown in recent studies,^92,93^ and this may depend on the cut-off of the cytokine storm, which may induce multiorgan dysfunction through excessive inflammatory mediators. Despite the high performance of adsorption ability for endotoxin and inflammatory mediators, there is no significant reduction of blood platelets during the Oxiris therapy process, which may rely on the pregrafted heparin allowing regional anti-coagulation on the surface of the filter. While no evidence remains that mortality is decreased among critically ill patients, Oxiris may be the bridge for stabilizing critically ill patients through improved hemodynamics and organ function before more conclusive therapies are taken.

### Novel devices

Apart from clearance of small molecular solutes, the kidney also presents metabolic, endocrinologic, and immunologic functions during sepsis. Based on this concept, renal cell therapy was developed using an extracorporeal device layered with renal tubule cells, and tested in preclinical animal models^94–98^ and FDA-approved multi-center human trials.^99^ Therapeutic benefits were observed in renal assist device (RAD) groups, but the development was suspended because of manufacturing and distribution issues. A selective cytopheretic inhibitory device (SCD) is another synthetic membrane device, which binds and deactivates neutrophils and monocytes during inflammation. SCD + CRRT treatment improved mortality and reduced dialysis dependency in a small-scale single-arm pilot study^100^ and a multi-center RCT carried out by the same research team.^101^ The bio-spleen is a blood-cleansing device for sepsis therapy inspired by the spleen. A broad spectrum of pathogens and toxins can be removed continuously without first requiring identification, providing more time to patients and researchers when facing attacks from unknown pathogenic microorganisms or toxins.^102^ More studies are required to examine any potential therapeutic benefits from all the promising novel devices mentioned.

## Side effects

It is important to bear in mind that all techniques will have side effects. As antibiotics are the mainstay of sepsis treatment, clinicians should be vigilant for unwanted antibiotic removal and under-dosing of patients. Inadequate levels of nutrients resulting from renal replacement therapy (RRT) (e.g. albumin leakage in HCO modality), and precise monitoring of therapeutic substance levels, particularly in critically ill patients, should be taken into account. Besides, the risks of hemorrhage, electrolyte imbalances, and catheter complications are similar to those of all other extracorporeal circuit techniques. Overall, frequent monitoring and appropriate adjustment through multi-disciplinary team (MDT) cooperation is strongly recommended, and crosstalk among nephrologists, critical care specialists, nurses, pharmacists, and nutritionists may advance management approaches for septic patients.

## Future direction

### Initiation timing

The optimal initiation timing of RRT for sepsis remains a point of discussion. The universally accepted indications (refractive acidosis, intense hyperkalemia, uremia, oliguria, and volume overwhelm unresponsive to diuretic therapy) for RRT in patients with AKI patients may not be applicable for patients with sepsis, considering some sepsis cases take place without advanced stage AKI. This requires clinicians to make a personalized therapeutic strategy for each case.

Early RRT could limit fluid excesses and organ damage, and potentially limit unbalanced host immune response in the septic patients. In an early application subgroup (within 3 hours sufficient fluid revitalization), a clinical trial in 15 patients with septic shock showed favorable results (reduction of vasopressor use, SOFA ranking, and increased survival) compared with the delayed application subgroup (initiation after organ damage had begun as a last-resort option).^103^ Another study reported reduced occurrence of sepsis and mortality in early HVHF (65 ml/kg/h 3 consecutive days after burning) patients from early de-cytokine clearance and patient immune recovery.^37^

Nevertheless, some studies have shown harmful effects of CRRT applied too early in septic patients, requiring clinicians to be careful.^104^ With early initiation of CRRT, patients still have sufficient renal function, and in addition to harmful antibiotics or nutrients, may be exposed to excessive circulation. According to the latest “Standard versus Accelerated Initiation of Acute Kidney Injury Renal Replacement Therapy" (STARRT-AKI, NCT02568722), the acceleration approach was not correlated with a lower risk of death in 90 days compared with the standard strategy for severely diseased AKI patients, but with substantial psychotherapy and bleeding incidents.^105^ This result is consistent with another recent trial focusing on sepsis.^106^

### Modality choice recommendation under the concept of precision medicine

As publications are currently scarce and inconsistent, existing guidelines on sepsis and septic shock do not include any guidance on the option of blood purification modality. Some “negative” outcomes may be the result of the genetic or immune profile of the patients enrolled, unsuitable indication, modality, dosage or duration choices. Therefore, future clinical trials should select patients more carefully to avoid such bias and in a more customized way, to ensure that they have the best therapy.

**Figure 1.**
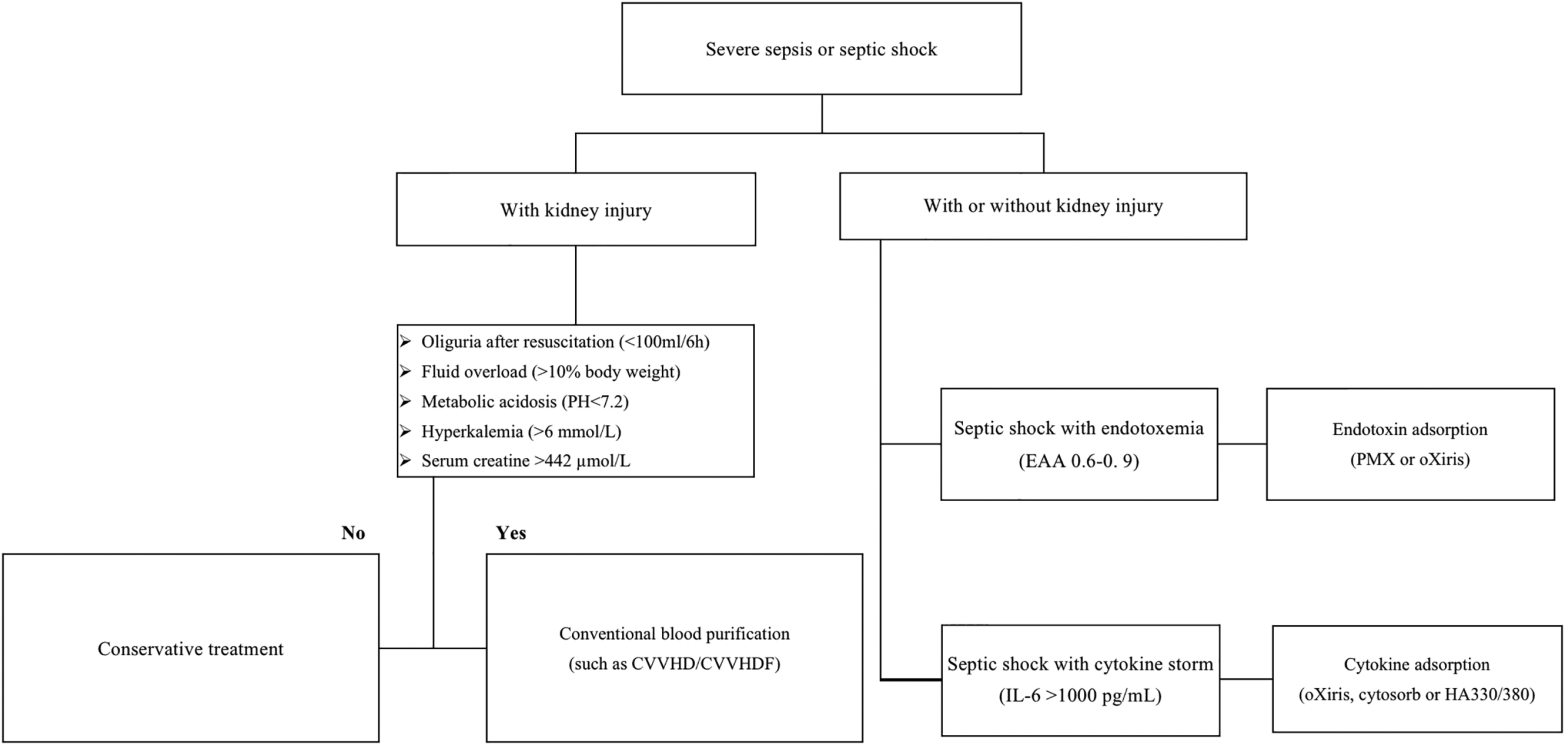
Decision-making strategy. EAA, endotoxin activity assay; PMX, polymyxin B-immobilized fiber; CVVHD, continuous veno-venous hemodialysis; CVVHDF, continuous veno-venous hemodiafiltration; IL-6, interleukin.

We want to share some personal opinion on the modality option of extracorporeal therapies in sepsis on the basis of the literature we reviewed. As shown in Fig. 1, after critically ill patient admission, with the help of SOFA, clinicians can make a diagnosis of sepsis/septic shock upon first response. For patients with sepsis who require additional RRT or those with septic shock, patients with/without AKI, adjuvant extracorporeal blood purification may kick in. First of all, the clinician can select the most beneficial modality for the patients based on the severity of sepsis and the endotoxin level. For patients at an early stage of sepsis, the application of adsorption/CPFA may be more beneficial by decreasing the endotoxin and cytokine peak levels with its wide clearance range of molecular weight compared with other techniques. As suggested by Klein *et al*., patients with an assay of endotoxin production ≥0.6–0.89 benefit more from endotoxin adsorption with Toraymyxin®.^62^ Furthermore, considering that the numerous positive results with the Polymyxin B-immobilized fiber column were obtained in Japan, and not replicated in two later trials conducted in Europe,^58,61^ we could hypothesize that a patient's genetic and enzymatic profile has a role in the patient's response to blood purification therapy. Pairing biomarkers for modality decisions may be an approach for future trials. Besides, a variety of other factors, including local infrastructure, RRT experience, nursing workload, and patient financial burden, should be taken into account in the final decision. To sum up, treatment should be adapted to the situation of the particular patient.

## Conclusion

Although extracorporeal blood purification offers new potential therapeutic strategies, it is too early to say whether RRT should be part of standard sepsis management. Conflicting results on patient survival rate justify further trials on topics not limited to indications, choice of modality, initiation timing and duration, dosage, and monitoring biomarkers of extracorporeal blood purification for septic shock. There is inadequate information to suggest one strategy over the others and one membrane (Table 1). Personalized therapeutic strategy made by MDT crosstalk is recommended.

**Table 1.** Major publications for each technique.

Modality	Author (Trial name)	Year	Patients	Comparators	Results
HVHF	Joannes-Boyau *et al*.^34^IVOIRE	2013	137 septic shock patients with AKI for less than 24 h	HVHF at 70 ml/kg/h (*n* = 66) versus Standard-volume haemofiltration at 35 ml/kg/h (*n* = 71)	- No difference in 28-day mortality- No difference in ventilator-, RRT-, and vasopressor-free days, length of stay, hemodynamic and standard biologic parameters, severity score evolution
Cascade	Quenot *et al*.^40^	2015	60 septic shock patients	Cascade group : usual care plus HVHF (*n* = 29) versus Control group: usual care alone (*n* = 31)	- Higher RRT-free days in the Cascade group- No difference in 7-, 28-, 90-day mortality- No difference in vasopressor- or ventilator-free days
HCO membrane	Atan *et al*.^47^	2018	76 critically ill patients with AKI	CVVH-HCO (cutoff point of 100 kDa, *n* = 38) versus CVVH-Std (cutoff point of 30 kDa, *n* = 38)	- No difference in median norepinephrine-free time- No difference in mortality, serum albumin levels, IV albumin administration, duration of hemofiltration, duration of norepinephrine infusion, and filter life
CPFA	Livigni *et al*.^55^COMPACT-1	2014	192 septic shock patients	Usual care plus CPFA (*n* = 62) versus Usual care alone (conventional therapy plus two sessions of polymyxin B hemoperfusion (n = 62)	- Lower mortality in patients receiving the higher dose of CPFA- No difference in new organ failures and ICU-free days within 30 days
	COMPACT-2	Early terminated in 2017	Septic shock patients	High doses CPFA with AMPLYA™ (BELLCO ITALY): >0.20 l/kg/day of plasma	- Higher early mortality (72 h)
Polymyxin B-immobilized fiber column	Cruz *et al*.^57^EUPHAS	2009	64 septic shock patients	Conventional therapy plus two sessions of polymyxin B hemoperfusion (*n* = 34) versus Conventional therapy (*n* = 30)	- Higher mean arterial pressure- Lower vasopressor requirement- Higher PaO_2_/FIO_2_ ratio- Lower SOFA scores- Lower 28-day mortality
	Payen *et al*.^58^ABDOMIX	2015	232 septic shock patients	Conventional therapy plus two sessions of polymyxin B hemoperfusion (*n* = 119) versus Conventional therapy (*n* = 113)	- No difference in 28-, 90-day mortality- No difference in reduction in SOFA score from day 0 to day 7- No difference in cytokines concentration
	Dellinger *et al*.^61^EUPHRATES	2018	450 septic shock patients with endotoxin activity assay level of 0.60 or higher	Conventional therapy plus two sessions of polymyxin B hemoperfusion (*n* = 224) versus Conventional therapy (*n* = 226)	- No difference in 28-day mortality Post hoc analysis:- Higher hemodynamic parameters, ventilator- free days, and survival rate in patients with EAA 0.6–0.89
CytoSorb	Schadler *et al*.^74^	2017	97 septic shock patients	CytoSorb hemoperfusion (*n* = 47) versus No hemoperfusion (*n* = 50)	- No decrease in plasmatic IL-6 level- No difference in mortality
	Hawchar *et al*.^75^	2019	20 septic shock patients without the need for renal replacement therapy	CytoSorb hemoperfusion (*n* = 10) versus No hemoperfusion (*n* = 10)	- Lower norepinephrine requirements, PCT concentration and Big-endothelin-1 concentrations
	Friesecke *et al*.^78^	Ongoing	198 patients (135 sepsis patients)	CytoSorb hemoperfusion	- Lower IL-6 level- Lower observed mortality than predicted- No difference in SOFA score
Oxiris	Not available				

HVHF, high-volume hemofiltration; IVOIRE, high Volume in Intensive care randomized controlled trial; AKI, acute kidney injury; RRT, renal replacement therapy; HCO, high cut-off; CVVH-HCO, continuous veno-venous hemodialysis-high cut-off; CVVH-Std, continuous veno-venous hemodialysis-standard; CPFA, coupled plasma filtration and adsorption; COMPACT, combining plasma filtration and adsorption clinical trial; ICU, intensive care unit; EUPHAS, early use of polymyxin B hemoperfusion in abdominal septic shock; PaO_2_, Partial Pressure of O_2_; FIO_2_, fraction of inspiration O_2_; SOFA, sequential organ failure assessment; ABDOMIX, Effects of Hemoperfusion With a Polymyxin B Membrane in Peritonitis With Septic Shock; EUPHRATES, Evaluating the Use of Polymyxin B Hemoperfusion in a Randomized Controlled trial of Adults Treated for Endotoxemia and Septic Shock; EAA, endotoxin activity assay; IL-6, interleukin 6; PCT, procalcitonin.
